# Structure of a rare non-standard sequence k-turn bound by L7Ae protein

**DOI:** 10.1093/nar/gku087

**Published:** 2014-01-29

**Authors:** Lin Huang, David M.J. Lilley

**Affiliations:** Cancer Research UK Nucleic Acid Structure Research Group, MSI/WTB Complex, The University of Dundee, Dow Street, Dundee DD1 5EH, UK

## Abstract

Kt-23 from *Thelohania solenopsae* is a rare RNA kink turn (k-turn) where an adenine replaces the normal guanine at the 2n position. L7Ae is a member of a strongly conserved family of proteins that bind a range of k-turn structures in the ribosome, box C/D and H/ACA small nucleolar RNAs and U4 small nuclear RNA. We have solved the crystal structure of *T. solenopsae* Kt-23 RNA bound to *Archeoglobus fulgidus* L7Ae protein at a resolution of 2.95 Å. The protein binds in the major groove displayed on the outer face of the k-turn, in a manner similar to complexes with standard k-turn structures. The k-turn adopts a standard N3 class conformation, with a single hydrogen bond from A2b N6 to A2n N3. This contrasts with the structure of the same sequence located in the SAM-I riboswitch, where it adopts an N1 structure, showing the inherent plasticity of k-turn structure. This potentially can affect any tertiary interactions in which the RNA participates.

## INTRODUCTION

The kink turn (k-turn) is a structural motif found in most functional RNA species, including the ribosome (1), spliceosome (2), several riboswitches (3–7) and box C/D (8) and H/ACA (9) small nucleolar ribonucleoprotein species. k-turns introduce a tight kink into the axis of double-stranded RNA sections, with an included angle of ∼50°, and frequently mediate tertiary interactions in larger RNA molecules, exemplified by the SAM-I riboswitch (3,10). In addition, most k-turns are binding sites for specific proteins. These include the L7Ae family of proteins (11,12), a group of structurally conserved proteins that includes the yeast snu31p, human 15.5 kDa (13) and bacterial homologs such as YbxF (14). Thus, L7Ae family proteins are important in ribosome structure, spliceosome assembly, small nucleolar RNA-directed modification of RNA and have recently been shown to be a subunit of archaeal RNaseP (15).

**Figure 1.**
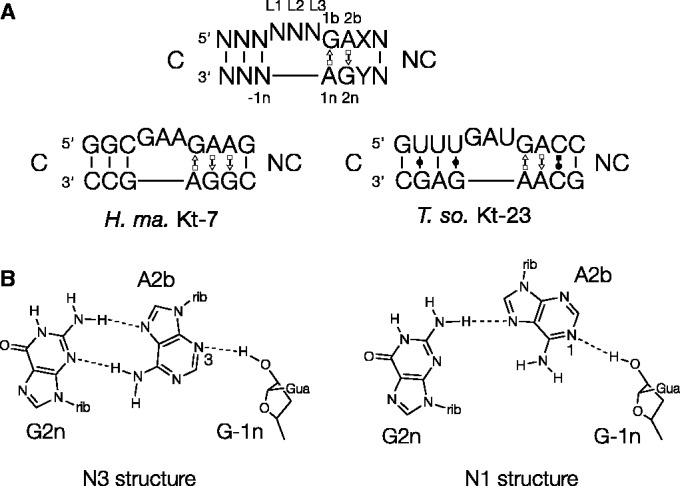
k-turn sequence and a key A-minor interaction. (**A**) The sequence of a standard simple k-turn, with the nomenclature (19) of key nucleotides indicated (top). The sequences of *H. marismortui* Kt-7 and *T. solenopsae* Kt-23 are shown below. (**B**) The two kinds of −1n O2′ to A2b cross-strand hydrogen bonds found in standard simple k-turns, accepted by either A2b N3 (left) or A2b N1 (right). This requires a change in rotational position of the A2b nucleobase, so that in the latter structure, usually the A2b N6 to G2n N3 distance is too long for stable hydrogen bonding.

In what we term the standard simple k-turn, a 3-nt bulge is followed on its 3′ side by G•A and A•G pairs, and frequently one further non-Watson–Crick pair (Figure 1A). This is exemplified by *Haloarcula marismortui* Kt-7. In its folded structure, the helical axis of the k-turn generates a tight kink. Folding can be promoted by the presence of metal ions (for some, not all, k-turns) (16), protein binding (17,18) or tertiary interactions (10). The structure is stabilized by a number of well-conserved hydrogen bonding interactions (19–21). Two key cross-strand interactions are accepted by the conserved adenine nucleobases in the G•A and A•G pairs, i.e. the 1n and 2b positions according to our nomenclature that is shown in Figure 1A (19). One of these is donated by the 2′-hydroxyl group of the nucleotide at the −1n position, and is accepted by the A2b nucleobase, either at N3 or N1 (Figure 1B). Virtually, all the known k-turns divide into two structural classes based on this distinction (21). The rotation of the A2b nucleobase required to accommodate hydrogen bonding at either N3 or N1 has a resulting effect on base pairing with A2n, so that only one hydrogen bond is possible in N1 class k-turns (21). Significantly, we have recently shown that Kt-7 can adopt either an N3 or N1 structure depending on its environment. While it adopts the N1 structure in the ribosome, it forms an N3 structure when replacing the natural k-turn of the SAM-I riboswitch (21), or as a simple duplex RNA either free or bound to the L7Ae protein (22).

A significant fraction of k-turn sequences depart from the ideal sequence, and this extends to the important G•A pairs. We term these non-standard simple k-turns, and this is well exemplified by Kt-23 of the small ribosomal subunit. Although the two adenine nucleotides (1n and 2b) are well conserved, the nucleotide at the 2n position is most often U, and relatively rarely is G (23). The least common Kt-23 sequence has an A at the 2n position, potentially creating an A•A pair at the 2b•2n position. In an earlier study, we modified the natural k-turn of the SAM-I riboswitch to create a G2nA substitution, and solved its structure by X-ray crystallography (10). The RNA folded as a normal k-turn (thereby allowing the riboswitch to bind its *S*-adenosylmethionine ligand), and the 2b•2n A•A pair was isosteric with a conventional G•A pair at the corresponding position, forming a *trans* A2b(Hoogsteen)•A2n(sugar) base pair connected by a single hydrogen bond from A2b N6 to A2n N3. A2b N3 accepted a hydrogen bond from G-1n O2′, and thus the structure was an N3 class k-turn.

Although extremely rare, such k-turns with an adenine at the 2n position do exist naturally, and bioinformatics analysis revealed that Kt-23 of the fire ant parasite *Thelohania solenopsae* is one such (Figure 1). This sequence was engineered into the SAM-I riboswitch in place of its natural k-turn, and the structure solved by crystallography (24). This revealed that the k-turn adopted the conventional structure. The A2b•A2n pair could again be classified as a *trans* A2b(Hoogsteen)•A2n(sugar) base pair, but the cross-strand hydrogen bond from G-1n O2′ was now accepted by A2b N1. As a consequence of its rotational setting, the nucleobase of A2b was not hydrogen-bonded to A2n. Thus, Kt-23 in the context of the riboswitch adopted an N1-class structure.

For the standard k-turns, we have shown that the switch between N3 and N1 structures leads to a change in axial rotation of the NC helix, which could have important consequences for making tertiary interactions (21). Therefore, an important question arises as to whether the non-standard *T. solenopsae* Kt-23 can change between N1 and N3 conformations depending on its environment in a manner similar to the standard k-turn Kt-7. The present work addresses this question.

In this study, we have used an alternative way to fold *T. solenopsae* Kt-23 into the k-turn conformation, *viz* protein binding, to see which structure is formed. We have previously shown that the binding of proteins can result in stable k-turn formation. For example, *Archeoglobus fulgidus* L7Ae binds to Kt-7 with high affinity (*K*_d_ = 10 pM) resulting in the formation of the kinked conformation (17,18). Single-molecule fluorescence resonance energy transfer (FRET) experiments are consistent with a conformational selection model for the folding process (25). A new crystal structure of the complex (22), together with earlier structures of complexes of L7Ae family proteins with different k-turns, shows that the manner of the interaction is largely structural recognition, and this is conserved in a wide range of complexes. Therefore, it is of considerable interest to see whether the structure of protein-bound Kt-23 remains N1 as it is in the SAM-I riboswitch, or whether it adopts the alternative structure. Therefore, we set out to crystallize a complex of *T. solenopsae* Kt-23 as a simple RNA duplex that is bound to the L7Ae protein. We find that the Kt-23 structure in the complex has adopted the N3 class structure, with a single hydrogen bond linking the A•A pair. This contrasts with the N1 structure adopted in the SAM-I riboswitch (24). Thus, the same Kt-23 sequence can switch between either the N1 or N3 structures depending on the environment, showing the plasticity of the *T. solenopsae* Kt-23 structure.

## MATERIALS AND METHODS

### RNA synthesis and construction of k-turn species

Ribooligonucleotides were synthesized using *tert*-butyldimethylsilyl phosphoramidite chemistry (26), as described in Wilson *et al.* (27). Oligoribonucleotides were deprotected in 25% ethanol/ammonia solution at 55°C for 2 h, and evaporated to dryness. Oligoribonucleotides were redissolved in 100-µl dimethyl sulfoxide to which 125 µl of 1 M triethylamine trihydrofluoride (Sigma-Aldrich) was added and incubated at 65°C for 2.5 h to remove *tert*-butyldimethylsilyl-protecting groups. All oligonucleotides were purified by gel electrophoresis in polyacrylamide in the presence of 7 M urea, and the full-length RNA product was visualized by ultraviolet shadowing. The band was excised and electroeluted using an Elutrap (Whatman) into 45 mM Tris borate (pH 8.5) and 5 mM ethylenediaminetetraacetic acid buffer for 8 h at 200 V at 4°C. The RNA was precipitated with ethanol, washed once with 70% ethanol and dissolved in water. The concentration of RNA was determined by measuring the absorbance at 260 nm using an extinction coefficient of 319.4 mM^−^^1 ^cm^−^^1^.

### Expression and purification of human U1 small nuclear ribonucleoprotein A

U1A-RBD (residues 1–102) (28) was expressed in the *Escherichia coli* BL21-Gold (DE3) pLysS cells (Stratagene) using a T7 RNA polymerase expression vector. The plasmid was generously provided by Dr K. Nagai (MRC-LMB, Cambridge, UK). Freshly transformed colonies were picked and grown in 6 L Luria broth (LB) medium, and then induced with 0.2 mM isopropyl β-d-1-thiogalactopyranoside at 37°C for 4 h. Harvested cells were resuspended in 20 mM Tris-HCl (pH 8.0), 50 mM NaCl, 1 mM phenylmethylsulfonyl fluoride (buffer T) and lysed by sonication. Cell debris was removed by centrifugation. U1A was loaded onto three tandem 5-ml CM columns (GE Healthcare), and the protein was eluted with 200 mM NaCl in buffer T. U1A was then applied to a heparin column (GE Healthcare) and eluted at 400 mM NaCl using a gradient from 50 to 2000 mM NaCl in 20 mM HEPES Na (pH 7.6). The protein was further purified using a Superdex 75 gel filtration column in a buffer containing 5 mM Tris-HCl (pH 8.0) and 100 mM NaCl.

### Expression and purification of *A. fulgidus* L7Ae

The gene encoding full-length *A. fulgidus* L7Ae was cloned into a modified pET-Duet1 plasmid (Novagen) (29) using the HindIII and EcoRI sites. The L7Ae gene was fused downstream of a hexahistidine-encoding sequence with a PreScission-cleavable linker. The hexahistidine–L7Ae fusion protein was expressed in *E. coli* BL21-Gold (DE3) pLysS cells (Stratagene) induced with 0.2 mM isopropyl β-D-1-thiogalactopyranoside at 20°C for 12 h.

Harvested cells were resuspended in 20 mM Tris-HCl, (pH 8.0), 500 mM NaCl, 10 mM imidazole, 1 mM phenylmethylsulfonyl fluoride (buffer A) and lysed by sonication. The protein suspension was heated at 85°C for 20 min in the presence of 10 mM MgCl_2_ to denature endogenous protein, and this was removed by centrifugation at 18 000 rpm for 30 min at 4°C. L7Ae was loaded onto a HisTrap column (GE Healthcare), washed with 25 mM imidazole in buffer A and the protein was eluted with 500 mM imidazole in buffer A. The His_6_ tag was cleaved from L7Ae by PreScission protease in 20 mM HEPES Na (pH 7.6), 100 mM NaCl and 0.5 mM ethylenediaminetetraacetic acid (buffer C) at 4–8°C for 16 h. L7Ae was applied to a heparin column (GE Healthcare) and eluted at 250 mM NaCl in a gradient from 50 to 2000 mM NaCl in 20 mM HEPES Na (pH 7.6). The protein was further purified using a Superdex 200 gel filtration column in a buffer containing 5 mM Tris-HCl (pH 8.0) and 100 mM NaCl.

The protein concentration was measured by absorbance at 280 nm using a molar extinction coefficient of 5240 M^−^^1 ^cm^−^^1^ for L7Ae. The protein was concentrated to 20 mg/ml in buffer containing 5 mM Tris-HCl (pH 8.0), 100 mM NaCl and stored at −20°C as aliquots.

### Crystallization, structure determination and refinement

A mixture of 0.25 mM RNA, 0.25 mM U1A-RBD and 0.25 mM L7Ae in 5 mM Tris-HCl (pH 8.0), 100 mM NaCl and 10 mM MgCl_2_ was incubated for 5 min at 37°C. Crystals were grown by vapor diffusion using drops prepared by mixing 1.0 μl of the RNA–protein complex with 1 μl of a reservoir solution comprising 100 mM Tris-HCl (pH 8.5) and 2.0 M ammonium dihydrogen phosphate at 7°C. Crystals (100–300 μm) appeared after 5 days. They were transferred to a solution containing 100 mM Tris-HCl (pH 8.4), 1.25 M ammonium dihydrogen phosphate, 3.15 M sodium formate for 25 h and then to 50 mM Tris-HCl (pH 8.4), 2 M ammonium dihydrogen phosphate, 30% glycerol for ∼10 s. Crystals were flash frozen by mounting in nylon loops and plunging into liquid nitrogen. They were characterized in-house with a MicroMax HF007 copper rotating anode X-ray generator equipped with an ACTOR sample changer system and a Saturn 944HG + CCD detector (Rigaku). Suitable crystals were stored and subsequently used to measure full data sets on beamline ID23-1 at the European Synchrotron Radiation Facility, Grenoble, France.

The crystals had space group C222_1_ and unit cell dimensions *a* = 135.9 Å, *b* = 155.5 Å and *c* = 146.9 Å. From crystal density considerations (30,31), two RNA–protein complexes were expected to be present in the asymmetric unit.

The structure was determined by molecular replacement. In the first stage, U1A protein plus the RNA binding loop with a 3-bp stem was used as the search model, followed by L7Ae protein, using the program PHASER (32). Inspection of residual electron density revealed the presence of two additional U1A-RBDs, and on inclusion of these, *R*_free_ reduced smoothly to 30% during refinement. The resulting electron-density maps revealed the remaining RNA density, and were built *de novo* on the basis of the difference map. Structural models were built in Coot (33) and refined with Refmac5 (34) from the CCP4 suite of programs (35) and Phenix (36). Omit maps were calculated using Phenix. Ramachandran analysis shows that 99.2% of amino acid residues are in the most favored and additionally allowed regions. Model geometry and the fit to electron-density maps were monitored with MOLPROBITY (37) and the validation tools in Coot. Atomic coordinates and structure factor amplitudes have been deposited with the PDB with accession code 4C4W.

## RESULTS

### Crystallization of a complex of Kt-23 with L7Ae

**Figure 2.**
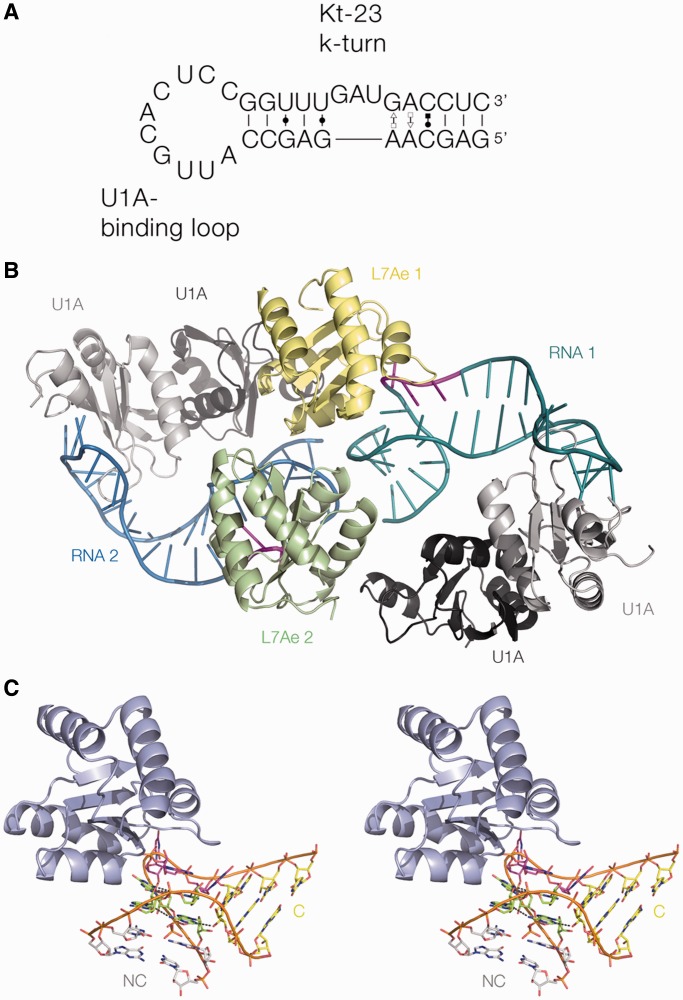
The structure of the Kt-23–L7Ae complex. (**A**) The sequence of the RNA species used in crystallization. It comprises a stem-loop, where the terminal loop is the binding site for the U1A protein. Kt-23 is centrally located in the duplex stem. (**B**) The structure of the two RNA-L7Ae-2.U1A complexes in the asymmetric unit. The two RNA molecules are shown in cartoon form, colored blue and green, with the loop nucleotides of Kt-23 highlighted in magenta. The L7Ae molecules are indicated in yellow and green. Two dimers of U1A (gray) are contained within the asymmetric unit, but only one monomer of each makes direct contact with the RNA terminal binding loop. (**C**) Parallel-eye stereoscopic image of the structure of one L7Ae–Kt-23 complex. The L7Ae protein is shown in cartoon form.

The RNA used in crystallization experiments comprised an 11-bp duplex in the center of which was the *T. solenopsae* Kt-23 sequence (Figure 2A). The duplex section terminated with a 10-nt loop that is the binding site for the U1A protein. The RNA was co-crystallized with a mixture of *Archeoglobus fulgidus* L7Ae and human U1A proteins. Orthorhombic crystals (space group C222_1_) were obtained that diffracted to 2.95 Å resolution, and the structure solved by molecular replacement using the structures of U1A and L7Ae. To avoid model bias, we have calculated a composite omit map, and the electron density presented here is derived from that. Details of data collection and refinement statistics for the crystallographic data are presented in Table 1.

### The overall structure

There are two complexes present in the asymmetric unit, each of which contains one fully base-paired RNA duplex bound by L7Ae in the center and a dimer of U1A of which one monomer is bound at the terminal loop (Figure 2B). The U1A protein and its target loop adopt their well-studied structures and manner of interaction (28,38,39). The L7Ae adopts the same fold observed in previous structures, such as the complex with Kt-7 (22), with a root-mean-square deviation = 0.53 Å for L7Ae 1. The overall nature of the interaction between L7Ae protein and the *T. solenopsae* Kt-23 k-turn (Figure 2C) is similar to that observed in its interaction with Kt-7 (22), the box C/D k-turn (8), as well as the homologous interaction between the human 15.5 kDa protein and the U4 small nuclear RNA k-turn (2). As in the other complexes, L7Ae binds in the continuous major groove that forms the outer face of the Kt-23 k-turn (Supplementary Figure S1). Two main regions of the protein interact with the RNA. These are the hydrophobic loop comprising residues 86–93 that envelops the L2 and L1 nucleobases, capping the NC and C helices, respectively, and a basic α-helix comprising residues 27–41. The details of the interaction will not be discussed further here, but additional data can be found in Supplementary Information.

### Structure of the Kt-23 k-turn

**Figure 3.**
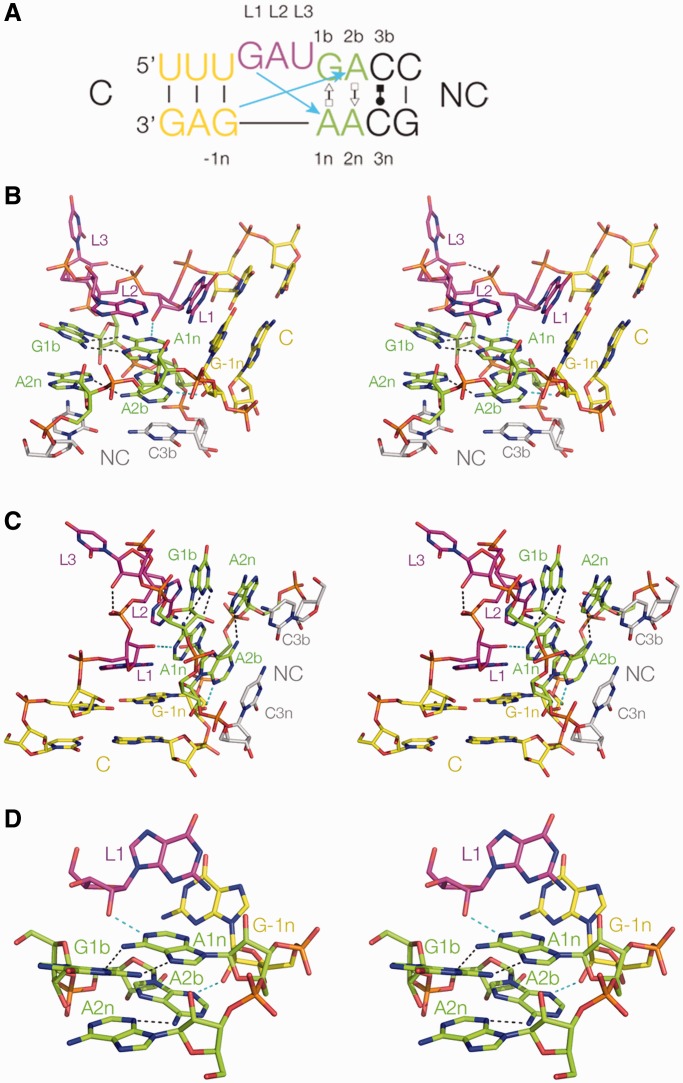
The structure of *T. solenopsae* Kt-23 contained within the complex with L7Ae. (**A**) The central sequence and base pairing of Kt-23 colored to correspond with that used in the molecular graphics images. The cyan arrows denote the two key cross-strand hydrogen bonds in the core of the structure. (**B–D**) Parallel-eye stereoscopic images of the Kt-23 structure, with hydrogen bonding denoted by broken lines. The key hydrogen bonds from L1 O2′ to A1n N1 and from −1n O2′ to A2b N3 are highlighted in cyan. (B) and (C) show views onto the non-bulged strand and bulge-containing strand side, respectively. (D) presents a closer view of the core of the structure. The A2b•A2n pair is also shown in Figure 4A.

Despite the presence of the rare A•A pair at the 2b•2n position, the *T. solenopsae* Kt-23 structure adopts conventional k-turn conformation in most respects (Figure 3) and superimposes with that of Kt-7 (22) with a root-mean-square deviation = 0.63 Å. In the complex with L7Ae, the L1 nucleobase is stacked onto the C helix, L2 in a *syn* conformation is stacked onto the NC helix and L3 is directed outward. The G•A pair at the 1b•1n position is a conventional *trans* G (sugar)•A (Hoogsteen) pair, buttressed by an additional cross-strand hydrogen bond between G1b N2 and A2n O2′ (N–O distance = 3.1 Å). The A•A pair at the 2b•2n position is connected by a single hydrogen bond from A2b N6 to A2n N3 (N–N = 3.0 Å).

We have previously shown that the nucleotides present at the 3b and 3n positions can be important in determining the stability of k-turns that do not have A•G at the 2b•2n position (23). In the *T. solenopsae* Kt-23 structure, these are both cytosine nucleotides, so there is the potential to make a C•C base pair. This is discussed further below. Beyond this, regular Watson–Crick pairing resumes, with a Watson–Crick C–G pair at 4b•4n. The C helix is conventionally double-stranded with Watson–Crick and U•G base pairs.

### Cross-strand hydrogen bonding

**Figure 4.**
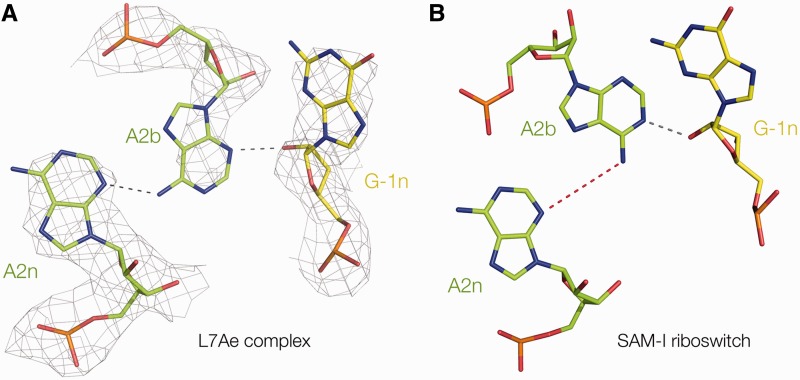
Comparison of the G-1n to A2b hydrogen bonding and A2n•A2b pairing in *T. solenopsae* Kt-23 structures. The sequence of the k-turn is identical in the two structures. (**A**) The structure of the A2n•A2b:-G-1n interaction in the complex with L7Ae. The electron density from the composite omit map is shown, contoured at 2σ. This is an N3 class structure in which G-1n O2′ donates its proton to N3 of A2b. The rotational setting of A2b allows its N6 to donate a proton to A2n N3. (**B**) The structure of the A2n•A2b:-G-1n interaction previously observed in the *T. solenopsae* Kt-23 structure inserted into the SAM-I riboswitch (24), in the absence of protein binding. This is an N1-class structure in which G-1n O2′ donates its proton to N1 of A2b. The required rotation of the nucleobase of A2b is incompatible with hydrogen bonding to A2n, so that the N–N distance of A2b N6 to A2n N3 is 4 Å in length (drawn red).

*T. solenopsae* Kt-23 has the standard cross-strand A-minor hydrogen bonds (19–21), donated from L1 O2′ and −1n O2′ to the nucleobases of the conserved adenine nucleotides at 1n and 2b, respectively. In addition, there is the near-universal hydrogen bond that closes the loop, from L3 O2′ to P1/P2 O *pro*S (O–O = 2.6 Å). All these hydrogen bonds have acceptable geometry, and the L1 O2′–A1n N1 bond has an O–N distance = 2.7 Å. The bond from −1n O2′ is accepted by A2b N3 (O–N = 2.7 Å) (Figure 4A), and thus the structure falls into the N3 class of k-turn structures (21). In common with N3 k-turn structures with a standard A2b•G2n base pair, the rotational setting of the A2b nucleobase allows a single hydrogen bond to form, from A2b N6 to A2n N3 (N–N = 3 Å). The structure of Kt-23 in the complex contrasts with that found when inserted into the SAM-I riboswitch, where it is an N1 structure lacking a hydrogen bond between A2b and A2n (24) (Figure 4B).

### The structure at the 3b•3n position

The 3b and 3n cytosine nucleobases are almost co-planar in the protein-bound Kt-23, as they are in the structure adopted in the SAM-I riboswitch (24). However, the C•C pairs are not identical in the two structures, showing a significant accommodation of the change in conformation between the N3 and N1 structures. At the resolution of the data, the electron density envelopes for the pyrimidine nucleobases are not as clearly defined as for those of purines, but the long axes can be placed with reasonable confidence. This indicates that there is a 3-Å translation of C3b relative to C3n between the two structures. In the N1 structure in the SAM-I riboswitch, the two cytosines are connected by a good hydrogen bond from C3b N4 to C3n O2 (N–O distance 2.9 Å and an angle of 131°). In the new structure, the probable orientation of the exocyclic amine and carbonyl would be inconsistent with the formation of a stable hydrogen bond.

**Table 1.** Details of data collection and refinement statistics for the crystallographic data as deposited in the PDB

Macromolecules	RNA-L7Ae-2.U1A complexes
PDB code	4C4W
Space group	C 2 2 21
Unit cell dimensions/Å	a = 135.9
	b = 155.5
	c = 146.9
	α = β = γ = 90.00°
**Molecules in the asymmetric unit**	
RNA-bound U1A	2
Free U1A	2
L7Ae	2
RNA	2
Ligands (2HP)	2
Resolution range/Å^a^	44.16–2.95
	(3.11–2.95)
Observations	216 474
Unique observations	31 598 (3068)
Completeness (%)	95.53 (93.74)
Mean((*I*)/σ_d_(*I*))	12.9 (2.0)
*R_merge_* (%)	10.7 (75.4)
CC(1/2)^b^	0.996(0.637)
Multiplicity	6.8 (5.3)
**Refinement statistics**	
Resolution range/Å	30.46–2.95
	(3.055–2.95)
R-factor	0.1670 (0.2820)
R-free	0.2107 (0.3163)
Number of atoms^c^	6364/10
Mean *B*-factor^d^ (Å^2^)	73.50/97.90
RMS bond length deviation/Å	0.008
RMS bond angle deviation/°	1.17
Ramachandran favored (%)	99.2

^a^Values in parentheses correspond to the highest resolution shell.

^b^Resolution cutoff criterion CC(1/2) > 0.6 according to (40).

^c^Number of atoms for macromolecules and ligands, respectively.

^d^Mean B-factors for macromolecules and ligands, respectively.

## DISCUSSION

We have presented a structure of the complex between a rare k-turn with the near-universal k-turn-binding protein L7Ae. L7Ae is a member of a widespread structurally conserved family of proteins that bind k-turns, important in the assembly of the ribosome, the box C/D and H/ACA nucleoproteins and the spliceosome. This study shows that the manner of protein–RNA recognition by this class of protein is conserved, and extends to non-standard k-turns such as *T. solenopsae* Kt-23. The non-specific interactions between the C-terminal end of the α-helix with the backbone of the non-bulged strand of the NC helix and the hydrophobic loop with the L1 and L2 loop nucleotides are found in all the complexes, and serve to measure the global shape of the k-turn. In addition, the N-terminal end of the α-helix makes a number of specific interactions with the strongly conserved G1b nucleobase in the major groove of the NC helix. In the complex between L7Ae and Kt-7, the side chain of N33 is hydrogen-bonded to G2n O6 (22), but the guanine is replaced by adenine in this structure. The side chain of N33 is difficult to locate in our electron density, suggesting that it is disordered. From its position, it could make an alternative interaction with A2n N6, but it seems unlikely that this interaction makes an important contribution to the stability of the complex. The majority of the interactions between L7Ae and this non-standard k-turn can be classed as structure selective for the folded conformation. This would explain the relatively broad spectrum of k-turns bound by L7Ae, and is consistent with our proposed conformational selection model for the protein-mediated folding process (25).

Kt-23 RNA of *T. solenopsae* is an unusual k-turn in that an adenine is in place of the normal guanine at the 2n position, thus creating a potential A•A pair at the 2b•2n position. The crystal structure shows that the *A. fulgidus* L7Ae protein binds to Kt-23 RNA of *T. solenopsae* in a manner closely similar to complexes with other k-turns, and that the RNA adopts a standard k-turn structure.

Comparison with our earlier structure of the same RNA sequence inserted into the SAM-I riboswitch (24) reveals a significant difference. In the new structure, where it is bound to L7Ae protein, the O2′ of G −1n is hydrogen-bonded to the N3 of A2b, whereas it was bonded to N1 of A2b in the SAM-I riboswitch context. As a consequence of the rotation of the A2b nucleobase, it forms a single hydrogen bond with A2n, from A2b N6 to A2n N3 (N–N = 3 Å) that is absent in the N1 class structure. Thus, precisely the same Kt-23 sequence can adopt an N3 or N1 class structure, depending on environment. Clearly, the complex of Kt-23 with L7Ae is not cognate, but the importance of the result is that the new structure demonstrates that this RNA can adopt the N3 k-turn conformation. We have previously shown that the near-consensus *H. marismortui* Kt-7 sequence can adopt either N1 (in the ribosome) or N3 [in the SAM-I riboswitch (21) or as a simple duplex either free or bound by L7Ae (22)] structures. Here we have a second example of this plasticity, for a non-standard simple k-turn with a sequence that significantly departs from the consensus.

Clearly, a number of environmental factors can influence the conformation adopted by a given k-turn, including local sequence, tertiary contacts and protein binding, and this evidently applies to *T. solenopsae* Kt-23. We have shown that in general the change between the N3 and N1 structures results in a change in axial rotation in the k-turn (21). The main difference between the environments in the SAM-I riboswitch and a simple duplex RNA is the loop-receptor interaction in the C helix of the former. This raises a further question because while *H. marismortui* Kt-7 adopts the N3 conformation in the riboswitch, Kt-23 in the same environment adopts the N1 structure. The probable reason for this lies in the C helix, where there are two U•G pairs in Kt-23. Examination of the structures shows that these introduce a reduced local twist angle, so that perhaps the switch from N3 to N1 compensates [the axial twist is larger for the N1 structure (21)] in order that the overall fit to the riboswitch structure is improved. This shows that the connections between local and larger-scale structure are subtle.

## ACCESSION NUMBERS

PDB code 4C4W.

## SUPPLEMENTARY DATA

Supplementary data are available at NAR Online.

## FUNDING

This work was funded by the Wellcome Trust and Cancer Research UK. Funding for open access charge: Wellcome Trust.

*Conflict of interest statement*. None declared.

## Supplementary Material

Supplementary Data
