# Supplementary material for: Structure of a rare non-standard sequence k-turn bound by L7Ae protein
Source: Nucleic Acids Res. 2014 Jan 29;42(7):4734–40. doi: 10.1093/nar/gku087 (PMC3985660; doi:10.1093/nar/gku087)
Supplement: Supplementary Data [file supp_42_7_4734__index.html]

Structure of a rare non-standard sequence k-turn bound by L7Ae protein — Structure of a rare non-standard sequence k-turn bound by L7Ae protein — Supplementary Data 

# Structure of a rare non-standard sequence k-turn bound by L7Ae protein

## Supplementary Data

files

**Files in this Data Supplement:**

- Supplementary Data - pdf file
