# Supplementary material for: Structure of a rare non-standard sequence k-turn bound by L7Ae protein
Source: Nucleic Acids Res. 2014 Jan 29;42(7):4734–40. doi: 10.1093/nar/gku087 (PMC3985660; doi:10.1093/nar/gku087)
Supplement: Supplementary Data [file supp_gku087_nar-03479-r-2013-File006.pdf]

Structure of a rare, non-standard sequence k-turn bound by L7Ae protein

Lin Huang and David M.J. Lilley

Supplementary Information

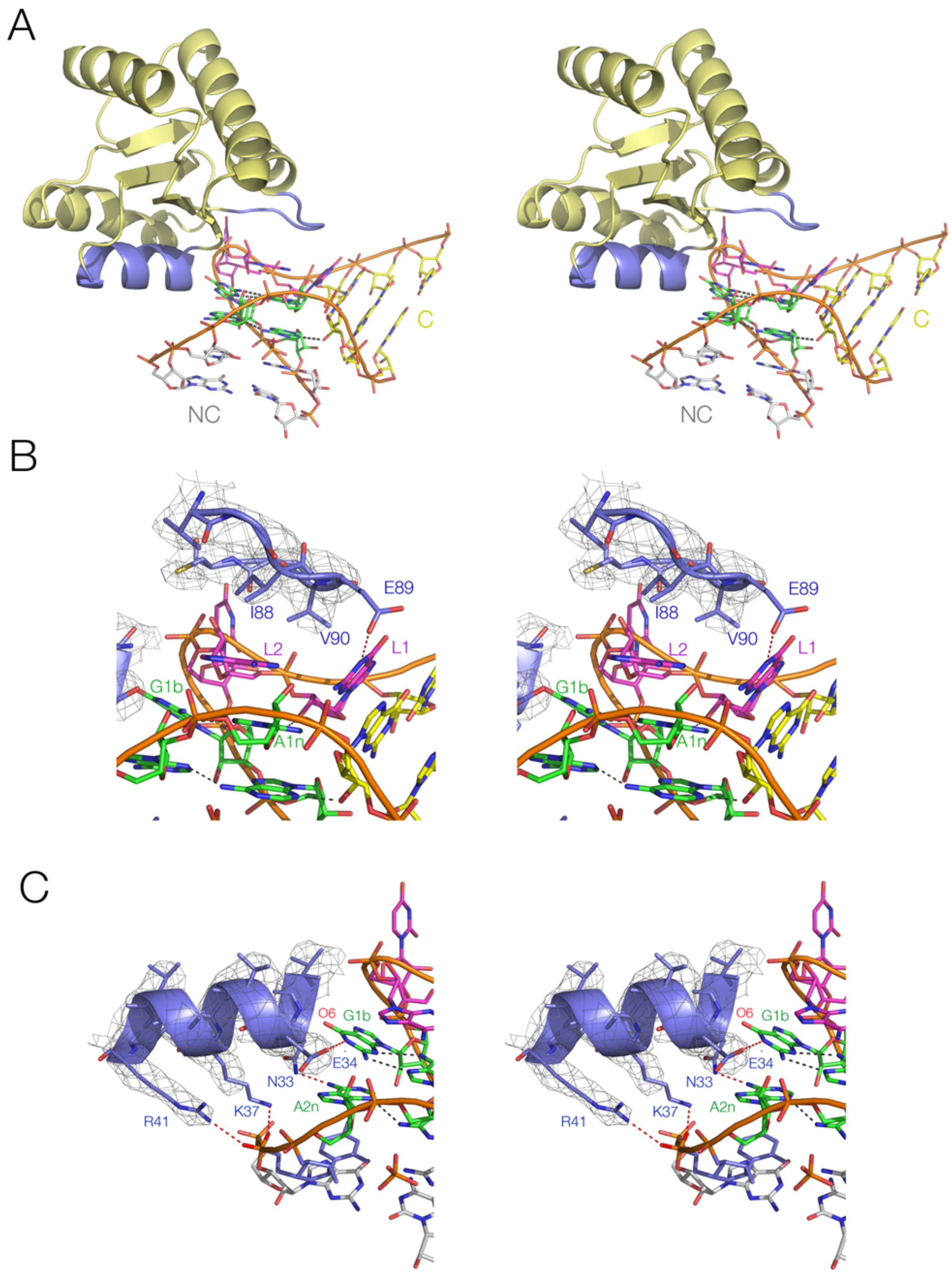

Figure S1. The interaction between L7Ae and Kt-23. Parallel-eye stereoscopic images of the complex.

A. A complete single L7Ae-Kt-23 complex. The L7Ae protein is shown in cartoon form,

with the key interacting elements of the N-terminal α-helix (residues 32-41) and the hydrophobic loop (residues 87-92) highlighted in blue.

B. The interaction between the hydrophobic loop and L1, L2 region. Note the hydrophobic side chains of I88 and V98 directed towards the RNA loop region, and the side chain of E89 that could hydrogen bond with L1.

C. The interaction between the α-helix and the major groove of the NC helix.

In parts **B** and **C**, hydrogen bonds between the protein and RNA are shown as broken red lines. The electron density from the composite omit map contoured at 2σ is shown for the protein.

The manner of the interaction between L7Ae protein and the *T. solenopsae* Kt-23 k-turn is very similar to that observed in its interaction with Kt-7 (1), the box C/D k-turn (2), as well as the homologous interaction between the human 15.5 kDa protein and the U4 snRNA k-turn (3). L7Ae binds in the continuous major groove that forms the outer face of the Kt-23 k-turn. Two main regions of the protein interact with the RNA, highlighted blue in Figure S1A. These are the hydrophobic loop comprising residues 86-93 that envelops the L2 and L1 nucleobases, capping the NC and C helices respectively, and a basic α helix comprising residues 27-41.

The hydrophobic loop has the sequence VGIEVPCA. It forms a kind of 'roof' that sits over the L2 nucleobase, and the L1 nucleobase capping the C helix (Figure S1B). The hydrocarbon side chains of isoleucine I88 and valine V90 are on the lower face, directed towards the RNA. There is good Van der Waals contact between the loop and both nucleobases, covering a total surface area of 732 $Å^2$. As seen in other L7Ae-k-turn complexes, the glutamate side chain (E89) is oriented towards the guanine L1 nucleobase where it could form a hydrogen bond, although it is not well defined by the omit map.

In L7Ae complexes with classic, simple k-turns such as Kt-7, the α helix makes both specific and non-specific interactions in the major groove of the NC helix (1). The α helix plus the preceding 4-residue β-sheet is overall very basic, having four lysine and one arginine residues. Figure 4C shows the location of this helix in the major groove of the NC helix of Kt-23, where the position of most of the side chains are well defined in the composite omit electron density map (Figure S1C). The position of this helix is reminiscent of the recognition helices of many DNA-binding proteins. Normally it is not possible to accommodate an α helix into the deep, narrow major groove of A-form duplex RNA, but the geometry of the k-turn 'splays out' the major groove on the outer face thus opening it up to such an interaction. The α helix makes a number of non-specific contacts with the backbone of the non-bulged strand of the

NC helix, in common with complexes with other k-turns. These include lysine K37 that is hydrogen bonded to the *pro*R non-bridging oxygen atom of the 3n/ 4n phosphate (N-O = 2.8 Å), and an arginine (R41) that bonded to the *pro*S non-bridging oxygen atom of the same phosphate (N-O = 3.1 Å). Lysine K28 (located on the preceding short β strand) also contributes to the general electronegative face of the protein juxtaposed with the NC helix.

The N-terminal end of the α helix is directed towards the Hoogsteen edges of the G1b and A2n nucleobases, making specific interactions that provide much of the specificity for the k-turn. The O6 atom of the conserved guanine at the 1b position (with a partial negative charge of ~ -0.6) is located at the positive pole of its helix dipole. In addition, the side chain of E34 (well defined in the omit map) is hydrogen bonded to the conserved G1b N1 (O-N distance = 3.3 Å). These interactions with G1b are preserved in all L7Ae-related protein complexes with k-turns such as the Kt-7 complex (1), the box C/D complex (2) and the complex of the 15.5 kDa protein with the U4 snRNA k-turn (3), and are clearly a very important part of the recognition of k-turn structure.

In standard k-turns, the nucleobase at the 2n position is a guanine, and in complexes such as that of Kt-7 this is recognized by the formation of a hydrogen bond from N33 to G2n O6 (1). However, in the *T. solenopsae* Kt-23 k-turn an adenine replaces the normal guanine at the 2n position. In the new structure N33 could form an alternative interaction with O6 of A2n (N-O distance = 2.7 Å); however the side chain is not well-defined by the composite omit electron density map, suggesting that this residue may be mobile and thus not involved in a tight interaction. It seems that the interaction with G1b is more important in k-turn recognition, and that substitution at the 2n position is tolerated more readily.

REFERENCES

1. Huang, L. and Lilley, D.M.J. (2013) The molecular recognition of kink turn structure by the L7Ae class of proteins. *RNA*, 19, 1703-1710.
2. Moore, T., Zhang, Y., Fenley, M.O. and Li, H. (2004) Molecular basis of box C/D RNA-protein Interactions; Cocrystal structure of archaeal L7Ae and a box C/D RNA. *Structure*, 12, 807-818.
3. Vidovic, I., Nottrott, S., Hartmuth, K., Luhrmann, R. and Ficner, R. (2000) Crystal structure of the spliceosomal 15.5 kD protein bound to a U4 snRNA fragment. *Molec. Cell*, 6, 1331-1342.
